# Phosphorylation of ERK5 on Thr732 Is Associated with ERK5 Nuclear Localization and ERK5-Dependent Transcription

**DOI:** 10.1371/journal.pone.0117914

**Published:** 2015-02-17

**Authors:** Takuto Honda, Yutaro Obara, Arata Yamauchi, Anthony D. Couvillon, Justin J. Mason, Kuniaki Ishii, Norimichi Nakahata

**Affiliations:** 1 Department of Cellular Signaling, Graduate School of Pharmaceutical Sciences, Tohoku University, 6–3 Aoba, Aramaki, Aoba-ku, Sendai 980–8578, Japan; 2 Department of Pharmacology, Yamagata University School of Medicine, 2–2–2 Iida-Nishi, Yamagata, 990–9585, Japan; 3 Cell Signaling Technology, 3 Trask Lane, Danvers, MA 01923, United States of America; Université de Sherbrooke, CANADA

## Abstract

Extracellular signal-regulated kinases (ERKs) play critical roles in numerous cellular processes, including proliferation and differentiation. ERK5 contains a kinase domain at the N-terminal, and the unique extended C-terminal includes multiple autophosphorylation sites that enhance ERK5-dependent transcription. However, the impact of phosphorylation at the various sites remain unclear. In this study, we examined the role of phosphorylation at the ERK5 C-terminal. We found that a constitutively active MEK5 mutant phosphorylated ERK5 at the TEY motif, resulting in the sequential autophosphorylation of multiple C-terminal residues, including Thr732 and Ser769/773/775. However, when ERK1/2 was selectively activated by an oncogenic RAS mutant, ERK5 phosphorylation at Thr732 was induced without affecting the phosphorylation status at TEY or Ser769/773/775. The Thr732 phosphorylation was U0126-sensitive and was observed in a kinase-dead mutant of ERK5 as well, suggesting that ERK1/2 can phosphorylate ERK5 at Thr732. This phosphorylation was also promoted by epidermal growth factor and nerve growth factor in HEK293 and PC12 cells, respectively. The ERK5–T732A mutant was localized in the cytosol under basal conditions. In contrast, ERK5 phosphorylated at Thr732 via the RAS-ERK1/2 pathway and ERK5–T732E, which mimics the phosphorylated form, were localized in both the nucleus and cytosol. Finally, ER–32A and U0126 blocked ERK5-dependent MEF2C transcriptional activity. Based on these findings, we propose a novel cross-talk mechanism in which ERK1/2, following activation by growth factor stimulation, phosphorylates ERK5 at Thr732. This phosphorylation event is responsible for ERK5 nuclear localization and ERK5-dependent transcription.

## Introduction

Extracellular signal-regulated kinases (ERKs), also called mitogen-activated protein kinases (MAPKs), participate in various cellular processes, including cell proliferation, differentiation, migration and gene expression. The MAPK family includes the classical MAPKs, such as ERK1/2, c-Jun N-terminal kinase 1/2/3, p38MAPK α/β/γ/δ and ERK5, as well as the atypical MAPKs ERK3, ERK4, ERK7 and nemo-like kinase (NLK) [1]. Threonine and tyrosine activation motifs (TXY) are conserved among all classical MAPKs and the atypical ERK7, whereas the other atypical MAPKs lack these motifs. ERK5 is approximately twice the molecular weight of ERK1/2. The kinase domain is encoded in its N-terminal half and shares approximately 50% homology with ERK1/2, while its unique C-terminal encodes two proline-rich regions and a nuclear localization signal and plays a critical role in transcriptional activation [2,3,4,5]. The threonine and tyrosine residues on ERK5 are specifically phosphorylated by the upstream kinase, MEK5. ERK5 is activated by a variety of stimuli, including growth factors [6,7,8], neurotrophic factors [9,10,11], cytokines [12] and stress [2,5], but the signaling pathways involved in ERK5 activation remain unclear. For example, the involvement of small G proteins such as RAS and RAP1 in ERK5 activation remains controversial [13], although it is well known that these small G proteins mediate ERK1/2 activation upon ligand binding to receptor tyrosine kinases [14,15].

ERK5 is physiologically essential, as demonstrated by a report showing that *ERK5* gene knockout is lethal at E9.5–10.5 because of cardiovascular defects [16]. These defects result from abnormal vasculogenesis and angiogenesis, and appear to arise from a primary endothelial cell defect rather than a myocyte abnormality [16,17]. Conditional deletion of *ERK5* in adult neurogenic regions involved in hippocampus-dependent memory formation impairs fear extinction, the expression of remote memory and olfactory behavior [18,19,20]. Furthermore, ERK5 plays critical roles in tumor development and cardiac hypertrophy [5,21,22].

We previously showed that ERK5 plays essential roles in neurite outgrowth, in the expression of the neurotransmitter synthesizing enzyme tyrosine hydroxylase in rat pheochromocytoma cells (PC12 cells) [11], and in expression of glial cell-derived neurotrophic factor in rat C6 glioma cells [6]. However, these effects were dependent on ERK1/2 as well, suggesting that both the ERK5 and ERK1/2 signaling cascades are necessary and that cross-talk between these pathways may occur. In a recent study, Morimoto et al. used deletion mutants of ERK5 consisting of the N-terminal (ERK5N) or the C-terminal (ERK5C) to clarify the role of specific phosphorylation sites on the protein [4]. In that study, multiple autophosphorylation sites on ERK5C were phosphorylated by an ERK5N mutant containing the kinase domain. An ERK5C mutant in which four of the autophosphorylation sites were substituted with aspartates enhanced the transcriptional activity of activator protein-1 (AP-1) and myocyte enhancer factor (MEF) 2. This finding suggests that ERK1/2 may phosphorylate these ERK5 autophosphorylation sites as well because ERK5N and ERK1/2 share substantial amino acid homology and their substrates largely overlap. In the present study, we investigated the interaction between ERK5 and ERK1/2 and examined whether ERK1/2 can phosphorylate the C-terminal of ERK5.

## Materials and Methods

### Materials

Nerve growth factor (NGF), epidermal growth factor (EGF) and luciferin were purchased from Sigma Aldrich (St. Louis, MO, USA). Antibodies against the phospho-ERK5 TEY motif (which cross-reacts with the phospho-ERK1/2 TEY motif), ERK5 and myc, horseradish peroxidase (HRP)-conjugated goat anti-rabbit IgG antibody, and U0126 were purchased from Cell Signaling Technology (Beverly, MA, USA). Anti-ERK2 antibody was purchased from Santa Cruz (Santa Cruz, CA, USA). Fetal bovine serum (FBS) was purchased from Cell Culture Laboratory (Cleveland, OH, USA). Lipofectamine 2000, horse serum and Alexa488-conjugated anti-rabbit IgG secondary antibody were purchased from Life Technologies (Grand Island, NY, USA). Polyvinylidene difluoride membrane, enhanced chemiluminescence (ECL) assay kits, hyper-film ECL and protein G sepharose beads were purchased from GE Healthcare (Little Chalfont, UK). Another ECL kit was purchased from PerkinElmer (Waltham, MA). GST-tagged ERK5 and ERK2 recombinant proteins were purchased from SignalChem (Richmond, Canada). A mycERK5’K83M kinase-dead mutant (KD), mycER–32A and mycER–32E were engineered from mycERK5 using the QuikChange site-directed mutagenesis kit (Stratagene, Cedar Creek, TX, USA). DNA plasmids encoding constitutively active MEK5 mutants (MEK5D, MEK5 (S311D/T315D)) were kindly provided by Dr. Eisuke Nishida (Kyoto University, Kyoto, Japan). DNA plasmids encoding mycERK2, constitutively active RAS-G12V (RASV12) and β-actin promoter-driven β-galactosidase were a kind gift from Dr. Philip J.S. Stork (Vollum Institute, Oregon Health & Science University, Portland, OR, USA). DNA plasmids encoding four × MEF2 response elements upstream of the minimal c-FOS promoter driving firefly luciferase (pMEF/Luc) were kindly provided by Dr. Ron Prywes (Columbia University, New York, NY, USA). Anti-phospho-Erk5 (pThr732 and pSer769/773/775) rabbit polyclonal antibodies were developed at Cell Signaling Technology in Danvers, MA (USA).

### Cell culture

HEK293 cells were grown in Dulbecco’s modified Eagle’s medium (DMEM) supplemented with 10% FBS, penicillin (50 units/ml) and streptomycin (50 μg/ml) in a 5% CO_2_ incubator at 37°C. PC12 cells were obtained from the Japanese Cancer Research Bank (Tokyo, Japan) and grown in DMEM supplemented with 10% FBS, 5% horse serum, penicillin (50 units/ml) and streptomycin (50 μg/ml) in a 5% CO_2_ incubator at 37°C.

### SDS-polyacrylamide gel electrophoresis and Western blotting

**Fig 1:**
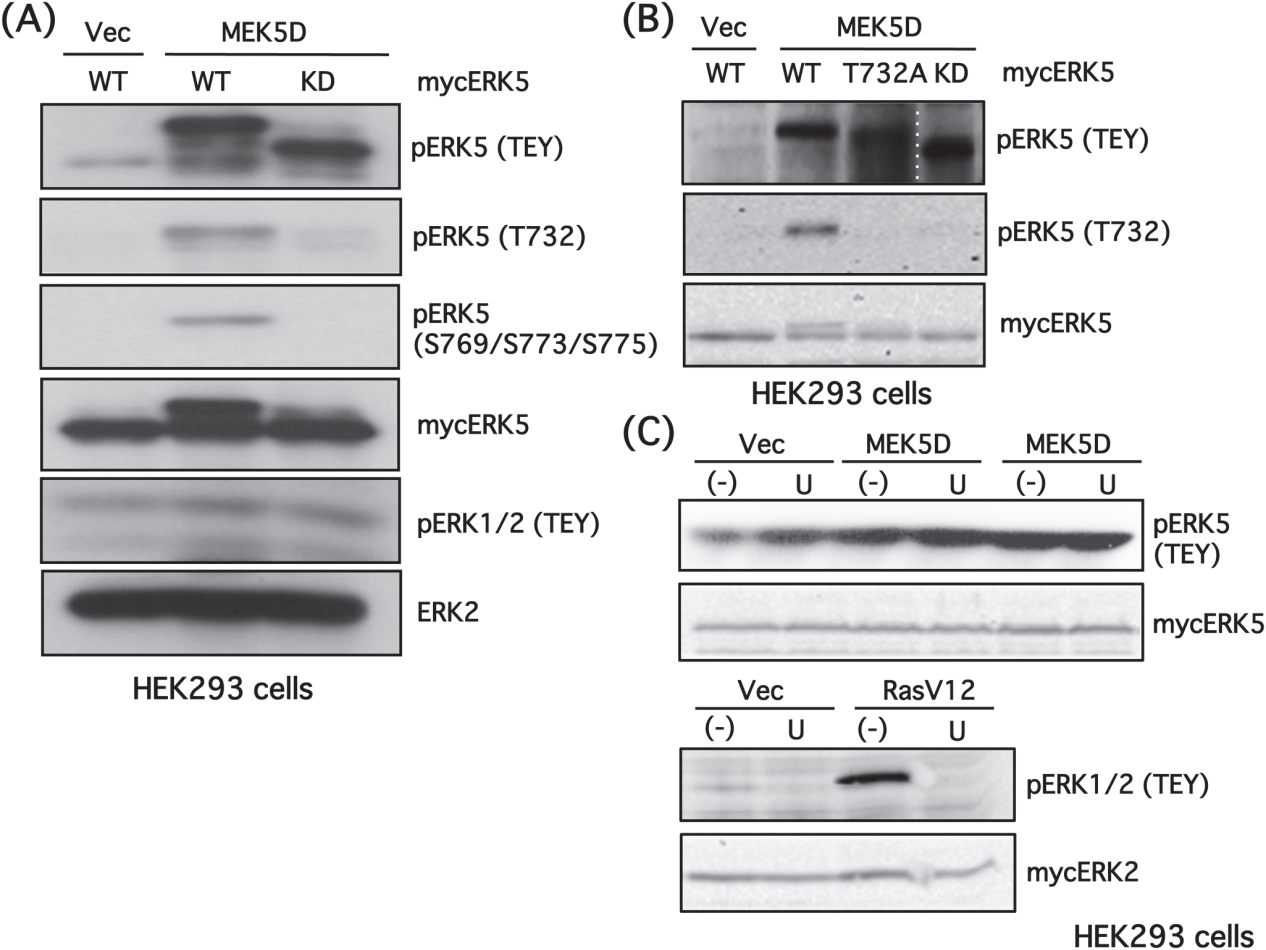
ERK5 Thr732 and Ser769/773/775 are autophosphorylated in HEK293 cells. **(A)** HEK293 cells were co-transfected with a constitutively active MEK5 mutant (MEK5D) and mycERK5 wild type (WT) or mycERK5 kinase-dead (KD) mutant. Empty vector (Vec) was used as a control. Cells were lysed, and ERK5 phosphorylation status at the TEY site, Thr732 and Ser769/Ser773/Ser775, as well as mycERK5, ERK1/2 phosphorylation at TEY, and ERK2 were examined by Western blotting. **(B)** HEK293 cells were co-transfected with MEK5D (or Vec) and mycERK5-WT, mycER–32A or mycERK5-KD. ERK5 phosphorylation status at the TEY site and Thr732, and mycERK5 were examined by Western blotting. **(C)** HEK293 cells were co-transfected with MEK5D (or Vec) (upper panels), constitutively active RAS mutant (RASV12) (or Vec) (lower panels) and mycERK5 (upper) or mycERK2 (lower). U0126 (30 μM) was added, and the phosphorylation status of ERK5 and ERK2 at their TEY sites and mycERK5 or mycERK2 were examined by Western blotting.

Cells or immunoprecipitates were dissolved in Laemmli sample buffer (final concentrations, 75 mM Tris-HCl, pH 6.8, 2% SDS, 15% glycerol, 3% 2-mercaptoethanol) and heated at 95°C for 5 min. Electrophoresis was performed on 8–11% polyacrylamide gels. Proteins were transferred onto polyvinylidene difluoride membranes using the semi-dry blotting method. The blots were blocked for 30 min with 5% skim milk in Tris-buffered saline supplemented with 0.1% Tween-20, and incubated with primary antibodies overnight at 4°C (1:1,000 dilution for antibodies against myc, phospho-ERK5 (pTEY), ERK5 and ERK2, and 1:500 dilution for antibodies against phospho-ERK5 (pThr732 or pSer769/S773/S775)). The blots were washed several times and then incubated at room temperature for 2 h with an HRP-conjugated secondary antibody (1:5,000 dilution). After washing, blots were developed using an ECL assay kit, and visualized by chemiluminescence on hyper-film ECL or with ChemiDocXRS (BioRad, Hercules, CA, USA). The densities of the bands corresponding to phospho-ERK5 at Thr732 were analyzed by densitometry (Image J64). The similar experiments were repeated at least three times, except for those in Figs. 1B and 1C, which were performed twice.

### Immunoprecipitation of endogenous ERK2

Briefly, cells were lysed in RIPA buffer containing protease inhibitors (1% Triton X-100, 1% sodium deoxycholate, 0.2% SDS, 125 mM NaCl, 50 mM Tris-HCl, 10% glycerol, 1 mM EDTA, 25 mM β-glycerophosphate, 1 mM PMSF, 10 μg/ml leupeptin, 10 μg/ml antipain, 10 μg/ml aprotinin, 2 μg/ml Na_3_VO_4_, pH 7.4). After sonication, the lysates were precleared with protein G sepharose beads for 1.5 h at 4°C. The precleared lysates were mixed with or without anti-ERK2 antibody (1:50) and 20 μl of protein G sepharose, and incubated for 2 h at 4°C in 500 μl RIPA buffer. The beads were washed twice and the immunoprecipitates were eluted by the addition of 25 μl Laemmli buffer. For examining *in vitro* interactions, ERK5 (0.5 μg) and ERK2 (0.5 μg) recombinant proteins were incubated in 500 μl of RIPA buffer for 30 min at 37°C. Then, after preclearing with protein G sepharose, the supernatants were immunoprecipitated with ERK2 antibody as described above.

### Immunofluorescence

HEK293 cells transfected with empty vector, mycERK5 wild-type (WT), mycER–32A or mycER–32E were fixed with 4% paraformaldehyde. In addition, cells co-transfected with mycERK5-WT or mycERK5-KD and oncogenic RASV12 mutant were also fixed with 4% paraformaldehyde. Cells were then treated with Triton X-100 (0.5%) and blocked with 5% bovine serum albumin for 2 h, and subsequently incubated with anti-myc primary antibody (1:400 dilution) at 4°C overnight followed by Alexa488-conjugated anti-rabbit IgG secondary antibody (1:300 dilution) for 3 h at 37°C. The nuclei were also stained with Hoechst 33258 (1 μg/ml). Then, cells were observed with a fluorescence microscope (Leica DMI3000B, Yamagata, Japan). The cell whose fluorescence intensity of the mycERK5-WT or the mutants in nucleus was similar or stronger than that in cytosol was defined as ERK5 nuclear localization, this ratio was counted in randomly selected areas.

### Reporter gene assay

For reporter gene assays, HEK293 cells were seeded into 24-well plates at a density of 0.5–1 × 10^5^ cells/well. The next day, DNA plasmids, including ERK5 (WT or mutant) and reporter genes (total 1 μg/well), were combined with Lipofectamine 2000 (1 μl/well) and mixed gently in serum-free DMEM (50 μl) and added to the culture plates for 4–6 h. Cells were then serum starved overnight and incubated with EGF in the presence or absence of U0126 at 37°C for 6 h. Cells were lysed in lysis buffer (1% Triton X-100, 110 mM K_2_HPO_4_, 15 mM KH_2_PO_4_, pH 7.8) (100 μl/well) and centrifuged to remove cell debris. The resulting supernatant (50 μl/tube) was mixed with 300 μl assay buffer (25 mM Gly-Gly, 15 mM MgSO_4_, 5 mM ATP, 10 mM NaOH). The luciferase reaction was started by the addition of 100 μl luciferin solution (150 μM), and luciferase activity was measured using a luminometer (LB9575; Berthold, Bad Wildbad, Germany). As an internal control, β-actin promoter-driven β-galactosidase activity was measured to normalize for transfection efficiency. The cell lysate (20 μl) was incubated with 100 μl reaction buffer (100 mM Hepes, 150 mM NaCl, 2 mM MgCl_2_, pH 7.0) supplemented with chlorophenol red-β-D-galactopyranoside (2 mg/ml) (Roche, Indianapolis, IN), and the absorbance at 595 nm was measured.

### Statistical analysis

Data were expressed as the mean ± SEM, and significant differences were assessed using the Tukey-Kramer method.

## Results

Upon phosphorylation of the ERK5 TEY activation motif by MEK5, multiple serine and threonine residues in the ERK5 C-terminal are autophosphorylated. It is thought that the C-terminal can, by itself, promote gene transcription when phosphorylated at Ser769/773/775 and Thr732 [4]. Thus, we hypothesized that ERK2 interacts with ERK5 and influences the phosphorylation status of the ERK5 C-terminal. To address this possibility, we created antibodies that recognize phospho-Ser769/773/775 or phospho-Thr732. In preliminary experiments, we showed that the phospho-Ser769/773/775 antibody displays the strongest preference for triple phospho-Ser peptides (data not shown). In HEK293 cells, constitutively active mutants of MEK5 (MEK5D) promoted ERK5 phosphorylation at its TEY site, resulting in sequential autophosphorylation at Thr732 and Ser769/773/775. A band shift of phospho-ERK5 was induced by the autophosphorylation, as often observed in other studies [11] (Fig. 1A). However, while MEK5D phosphorylated a kinase-dead mutant of ERK5 (ERK5-KD) at its TEY site, autophosphorylation at Thr732 and Ser769/773/775 and the associated band shift were no longer observed because of the absence of kinase activity (Fig. 1A). ERK1/2 phosphorylation was not induced by MEK5D.

To evaluate the specificity of the antibody against phospho-ERK5 at Thr732, ERK5-WT, ER–32A and ERK5-KD were expressed in HEK293 cells, and the phosphorylation status of these ERK5 mutants was examined. As expected, phosphorylation of ERK5 at Thr732 was diminished in ER–32A- and ERK5-KD-expressing cells (Fig. 1B), confirming the specificity of the antibody. The band shift of ERK5 was partially blocked in ER–32A-transfected cells. We also assessed the specificity of U0126, an inhibitor of ERK1/2 signaling, because the effects of U0126 on MEK5 activity have been controversial [23,24,25,26,27,28,29,30]. Although ERK5 phosphorylation at the TEY site by MEK5D was not affected by U0126 in HEK293 cells, ERK1/2 phosphorylation by oncogenic RAS signaling was abolished by U0126 (Fig. 1C). This result is consistent with our previous report [11] and the result in the present study showing that EGF and NGF-induced ERK5 phosphorylation at the TEY site is not blocked by U0126, whereas ERK1/2 phosphorylation is completely inhibited.

**Fig 2:**
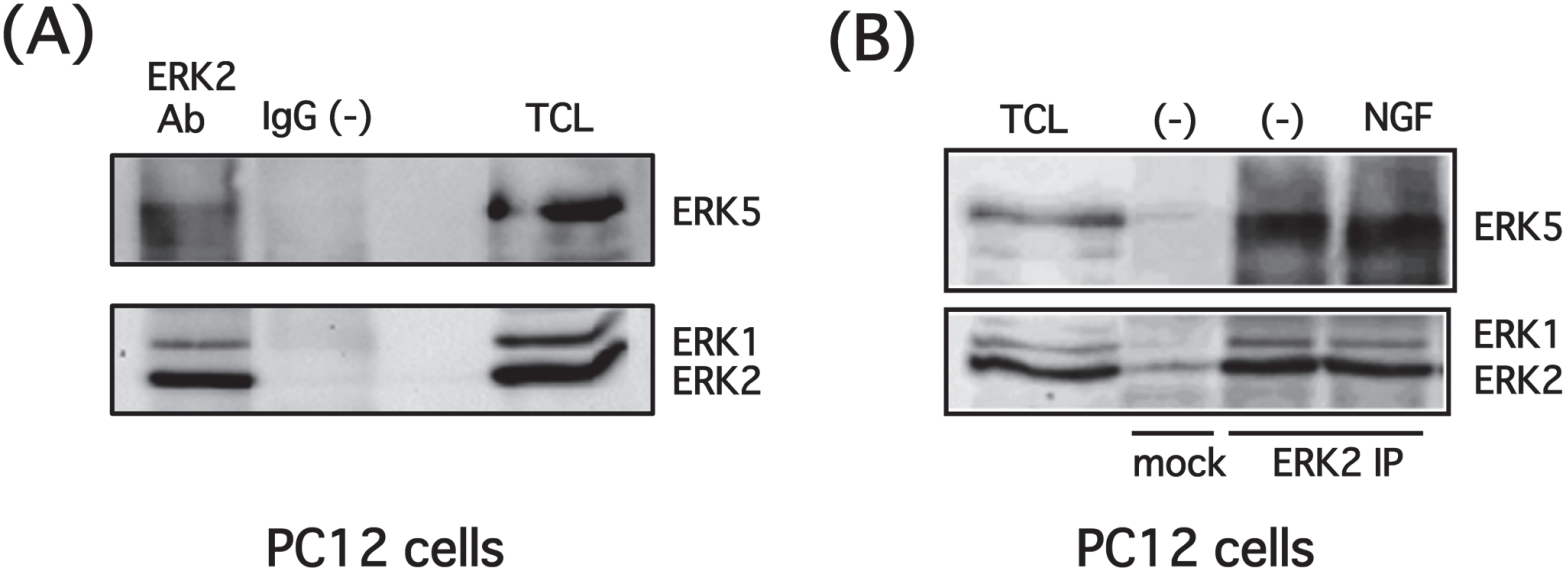
Endogenous ERK5 forms a complex with ERK1/2 in PC12 cells. **(A)** PC12 cells were lysed, and endogenous ERK2 was immunoprecipitated using an anti-ERK2 antibody. Endogenous ERK5 and ERK2 in the immunoprecipitates or total cell lysate (TCL) were examined by Western blotting. **(B)** PC12 cells were stimulated with NGF (100 ng/ml) for 5 min, then the cells were lysed, and endogenous ERK2 was immunoprecipitated using an anti-ERK2 antibody. Endogenous ERK5 and ERK2 in the immunoprecipitates or total cell lysate (TCL) were examined by Western blotting.

It has been shown that ERK1/2 forms homodimers upon phosphorylation by the upstream kinase MEK1/2 [31]. In addition, the ERK5 N-terminal domain is important for oligomerization, and ERK5 oligomerizes under both stimulated and unstimulated conditions, although the role of oligomerization remains unclear [5,32]. Because we sought to better understand the interaction between ERK5 and ERK1/2 and determine whether ERK1/2 affects ERK5 phosphorylation at the C-terminal, we examined if these ERKs also form heterodimers/oligomers. As expected, endogenous ERK5 co-immunoprecipitated with ERK2, indicating that endogenous ERK2 (possibly ERK1 as well) also complexes with ERK5 in PC12 cells (Fig. 2A). This complex did not change after NGF stimulation (100 ng/ml, 5 min) (Fig. 2B). To investigate whether this interaction between ERK5 and ERK2 is direct or indirect, we performed *in vitro* ERK5-ERK2 protein binding experiments using recombinant proteins. Proteins were co-incubated for 30 min at 37°C, and ERK2 was immunoprecipitated. ERK5 was absent in the immunoprecipitates under our experimental conditions, suggesting that ERK5 binds to ERK2 indirectly (data not shown).

**Fig 3:**
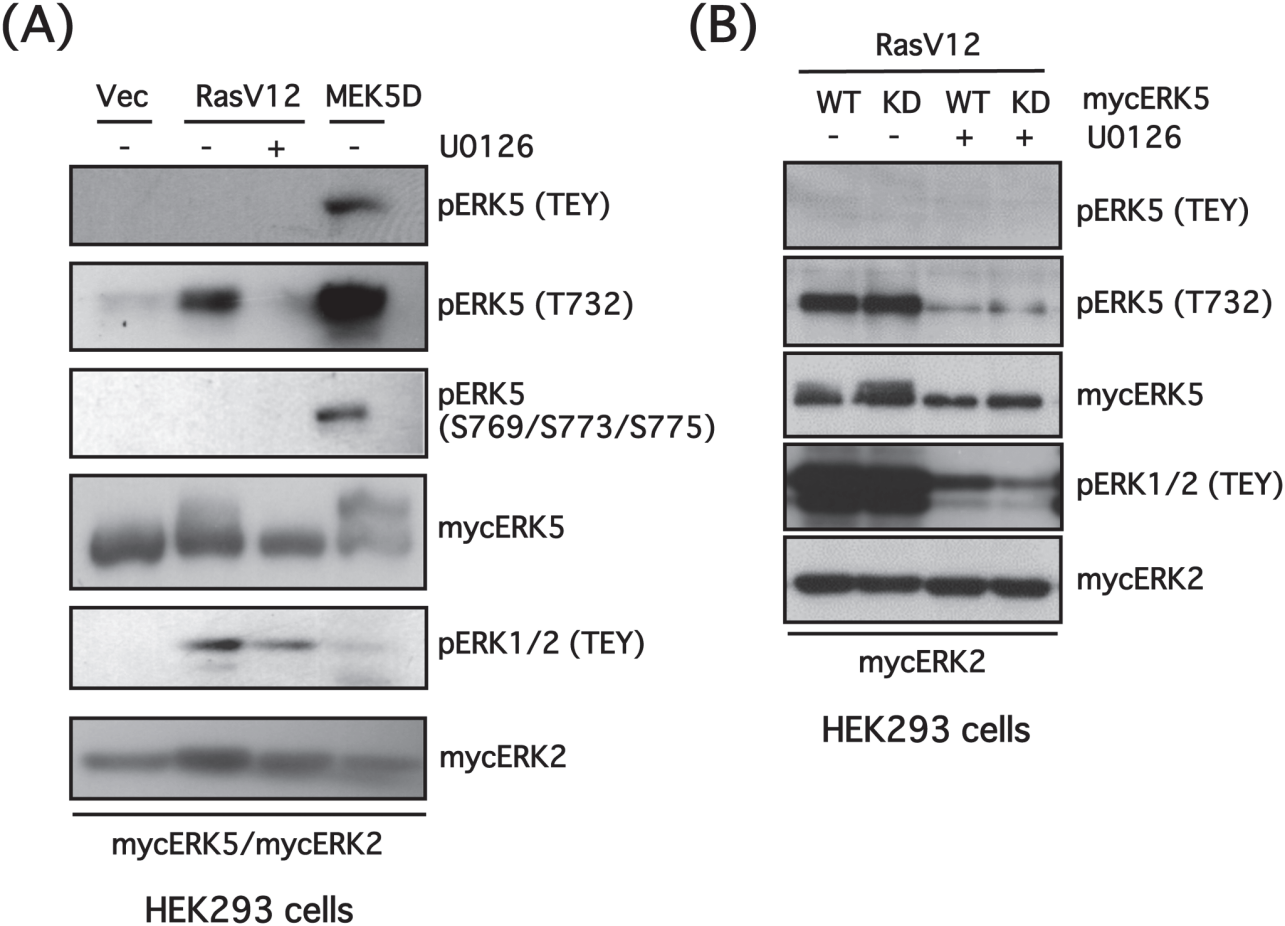
ERK1/2 phosphorylates ERK5 at Thr732 in HEK293 cells. **(A)** HEK293 cells were co-transfected with mycERK5, mycERK2 and empty vector (Vec), constitutively active RAS mutant (RASV12) or constitutively active MEK5 mutant (MEK5D). The cells were incubated in the presence or absence of U0126 (20 μM) for 24 h, then ERK5 phosphorylation status at its TEY site, Thr732 and Ser769/773/775, phospho-ERK1/2 (TEY), mycERK5 and mycERK2 were examined by Western blotting. **(B)** HEK293 cells were co-transfected with RASV12, mycERK2 and mycERK5 wild-type (WT) or mycERK5 kinase-dead (KD) mutant. The cells were incubated in the presence or absence of U0126 (20 μM) for 24 h, then ERK5 phosphorylation status at its TEY site and Thr732, phospho-ERK1/2 (TEY), mycERK5 and mycERK2 were examined by Western blotting.

We previously demonstrated that a constitutively active mutant of RAS (RASV12) promotes phosphorylation of the TEY site in ERK1/2, but not the TEY site of ERK5 in PC12 cells [11]. Furthermore, we showed that RASV12 caused phosphorylation of ERK1/2 at its TEY site, but not at the ERK5 TEY site in HEK293 cells. Nevertheless, phosphorylation of ERK5 at Thr732 was enhanced 2.7-fold, and was completely blocked by U0126 (Fig. 3A). The phosphorylation status of ERK5 at Ser769/773/775 was not altered by RASV12. Furthermore, RASV12 similarly promoted the phosphorylation of ERK5-KD at Thr732 in a U0126-sensitive manner (Fig. 3B), suggesting that phosphorylation of ERK5 at Thr732 is also induced by activated ERK1/2.

**Fig 4:**
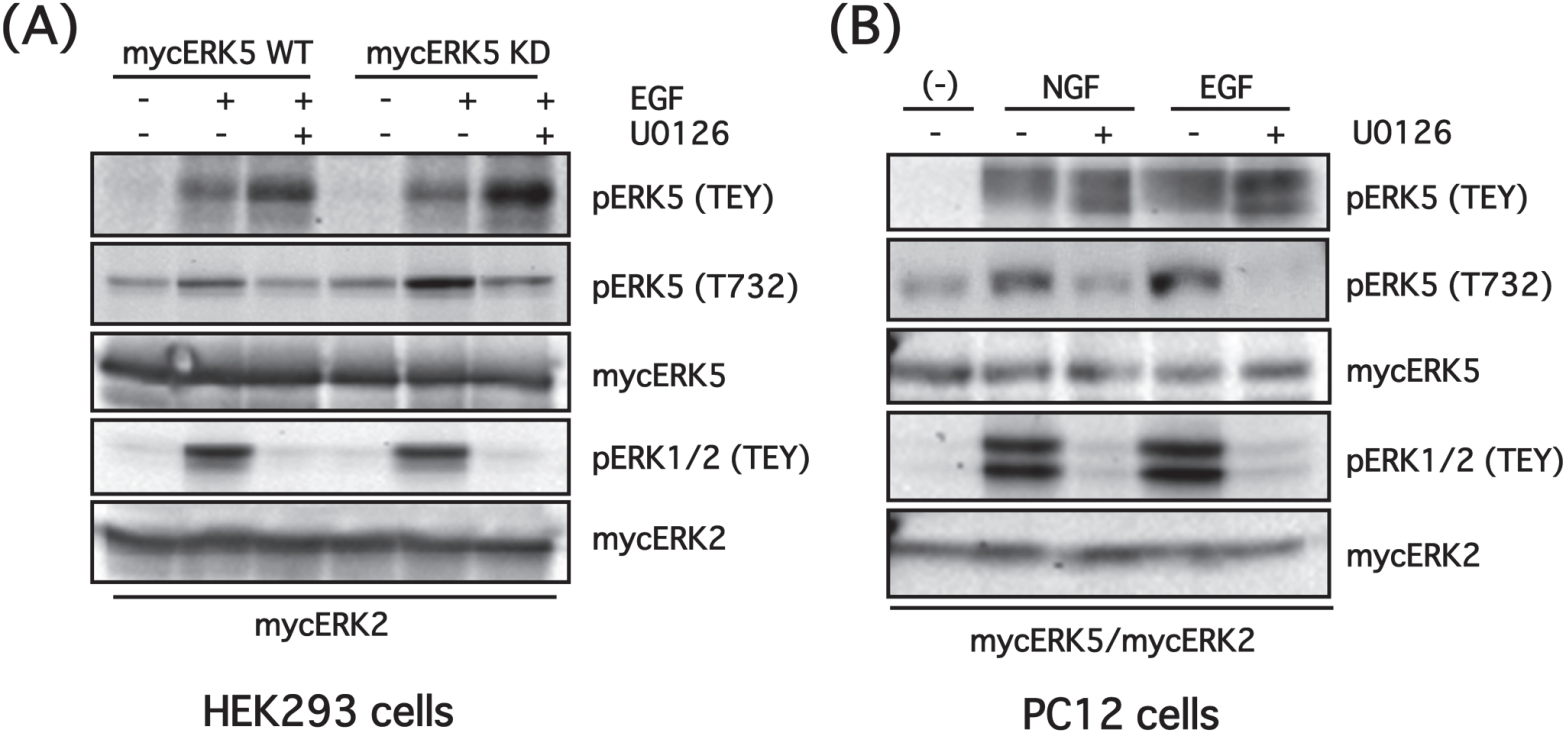
EGF and NGF promote ERK5 phosphorylation at Thr732 via ERK1/2 in HEK293 and PC12 cells. **(A)** HEK293 cells were co-transfected with mycERK2 and mycERK5 wild-type (WT) or mycERK5 kinase-dead (KD) mutant. After the cells were preincubated in the presence or absence of U0126 (20 μM) for 30 min, they were stimulated with EGF (100 ng/ml) for 5 min. Then, ERK5 phosphorylation status at its TEY site and Thr732, phospho-ERK1/2 (TEY), mycERK5 and mycERK2 were examined by Western blotting. **(B)** PC12 cells were co-transfected with mycERK5 and mycERK2. After the cells were preincubated in the presence or absence of U0126 (20 μM) for 30 min, they were stimulated with NGF (100 ng/ml) or EGF (100 ng/ml) for 5 min. Then, ERK5 phosphorylation status at its TEY site and Thr732, phospho-ERK1/2 (TEY), mycERK5 and mycERK2 were examined by Western blotting.

Because growth factors such as EGF or NGF promote phosphorylation of both ERK1/2 and ERK5 at their respective TEY sites, we examined the phosphorylation status of ERK5 at Thr732 when both ERK isoforms were physiologically activated by these growth factors. HEK293 cells were co-transfected with mycERK2 and mycERK5-WT or mycERK5-KD, then stimulated with EGF (100 ng/ml) for 5 min in the presence or absence of U0126 (20 μM). EGF induced phosphorylation of ERK5 at Thr732 1.60-fold in addition to phosphorylation at the TEY sites of both ERK1/2 and ERK5 in HEK293 cells. U0126 blocked phosphorylation of ERK1/2 at the TEY site and ERK5 at Thr732 (reducing phosphorylation status to approximately basal levels). ERK5-KD was also phosphorylated at both the TEY site and Thr732 (Fig. 4A). Experiments were also performed in PC12 cells co-transfected with mycERK2 and mycERK5-WT, then stimulated with NGF (100 ng/ml) or EGF (100 ng/ml) for 5 min in the presence or absence of U0126 (20 μM). As in HEK293 cells, both NGF and EGF caused ERK5 phosphorylation at Thr732 in an ERK1/2-dependent manner (Fig. 4B). These results strongly suggest that ERK5 Thr732 is preferentially phosphorylated by ERK1/2, but not by ERK5 itself, when both ERK isoforms are physiologically activated by growth factors. However, ERK5 can autophosphorylate ERK5 at Thr732 when ERK5 is strongly and selectively activated by MEK5D.

**Fig 5:**
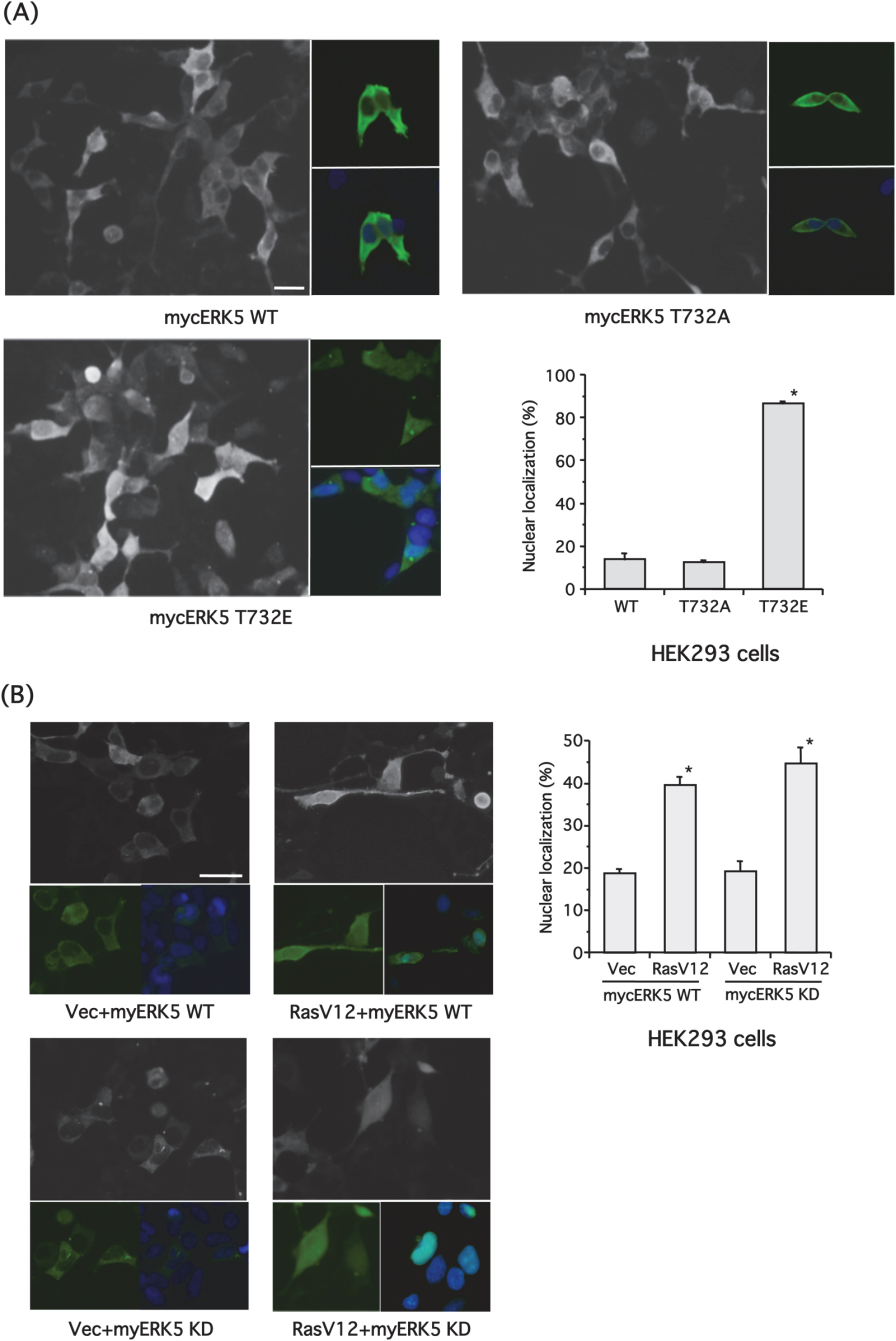
ERK5 phosphorylation at Thr732 via ERK1/2 is required for nuclear localization in HEK293 cells. **(A)** HEK293 cells were transfected with empty vector, mycERK5 wild-type (WT), mycER–32A or mycER–32E. The myc-tagged ERKs were stained with myc primary antibody and Alexa488-conjugated secondary antibody with Hoechst 33258. Scale bar = 25 μm. The ratio of nuclear localization was also examined. Values represent the means ± SEM. * *P* < 0.05 compared with WT (n = 3). **(B)** HEK293 cells were co-transfected with mycERK5-WT or mycERK5-KD and empty vector or RASV12. The myc-tagged ERKs were stained with myc primary antibody and Alexa488-conjugated secondary antibody with Hoechst 33258. Scale bar = 25 μm. The ratio of nuclear localization was also examined. Values represent the means ± SEM. *P < 0.05 compared with empty vector (n = 3).

Diaz-Rodriguez et al. examined the subcellular localization of ERK5-WT and its mutants, including those in which four Ser and Thr residues including Thr732 were substituted with Ala or Glu (i.e. 4xPA or 4xPE, respectively). While HA-tagged ERK5-WT and ERK5–4xPA maintained an essentially cytoplasmic distribution, the phospho-mimetic ERK5–4xPE had a nuclear localization [33]. In the present study, we used mycERK5-WT, mycER–32A and mycER–32E to examine the intracellular localization of ERK5. mycERK5-WT and mycER–32A mainly localized to the cytosol (Fig. 5A). In contrast, mycER–32E was localized in both nucleus and cytosol (Fig. 5A). There was no green fluorescence in cells transfected with empty vector alone (data not shown). Next, we examined the intracellular localization of mycERK5-WT and mycERK5-KD phosphorylated at Thr732 by RASV12-ERK1/2 signaling. RASV12-ERK1/2 signaling promoted retention of ERK5 in both nucleus and cytosol probably through phosphorylation at Thr732, as demonstrated in Fig. 3 (Fig. 5B). These results suggest that phosphorylation of Thr732 is important for ERK5 subcellular localization.

**Fig 6:**
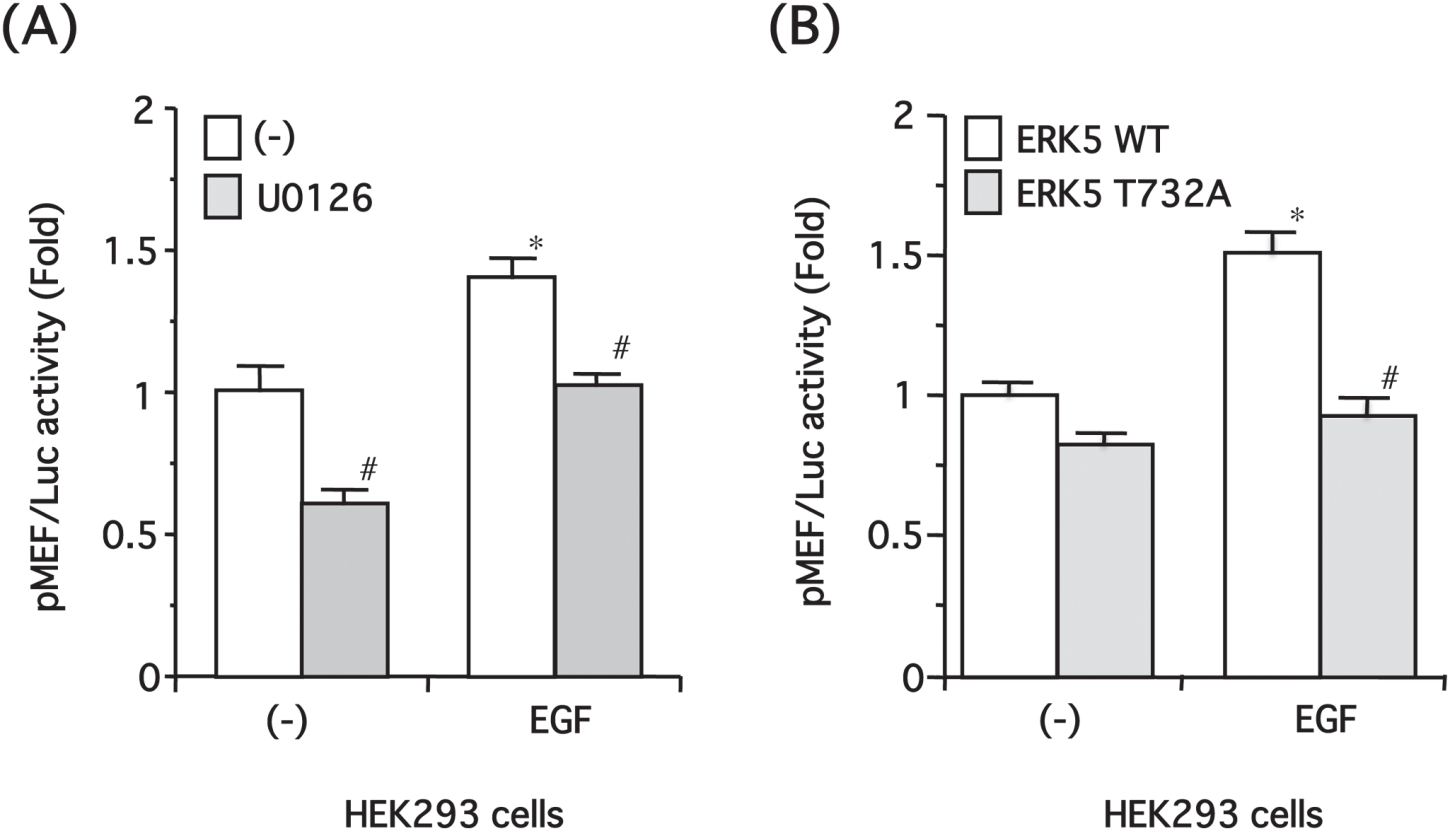
ERK5 phosphorylation at Thr732 via ERK1/2 is required for MEF2C-dependent transcription in HEK293 cells. **(A)** HEK293 cells were co-transfected with pMEF2/Luc and β-galactosidase driven by the β-actin promoter. The cells were incubated with EGF (100 ng/ml) in the presence or absence of U0126 (30 μM) for 6 h, and luciferase assays were performed. Values represent the means ± SEM. *P< 0.05 compared with (-) and ^#^ *P* < 0.05 compared with (-) or EGF alone (n = 6). **(B)** HEK293 cells were co-transfected with pMEF2/Luc, β-galactosidase driven by β-actin promoter, and mycERK5 wild-type (WT) or mycER–32A. The cells were incubated with EGF (100 ng/ml) for 6 h, and luciferase assays were performed. Values represent the means ± SEM. *P < 0.05 compared with (-) and ^#^ *P* < 0.05 compared with ERK5-WT (n = 6).

Because phosphorylation of the ERK5 C-terminal is thought to enhance gene transcription independently of the phosphorylation of transcription factors [4], we next examined the role of ERK1/2-mediated phosphorylation of ERK5 at Thr732 on MEF2C-dependent gene transcription. MEF2C is a transcription factor that is well known to be phosphorylated and activated by ERK5 [34]. HEK293 cells were transfected with a pMEF2/luciferase reporter gene, then stimulated with EGF (100 ng/ml) for 6 h in the presence or absence of U0126 (30 μM). EGF significantly increased MEF2 activity, and this effect of EGF was significantly reduced by U0126 (Fig. 6A). Next, HEK293 cells were co-transfected with pMEF2/luciferase and mycERK5-WT or mycERK-T732A, then stimulated with EGF (100 ng/ml) for 6 h. Although EGF significantly increased MEF2 activity in cells overexpressing ERK5-WT, this effect of EGF was significantly reduced in cells expressing ER–32A (Fig. 6B), suggesting that ERK1/2-dependent phosphorylation of ERK5 at Thr732 enhances MEF2C activation.

## Discussion

**Fig 7:**
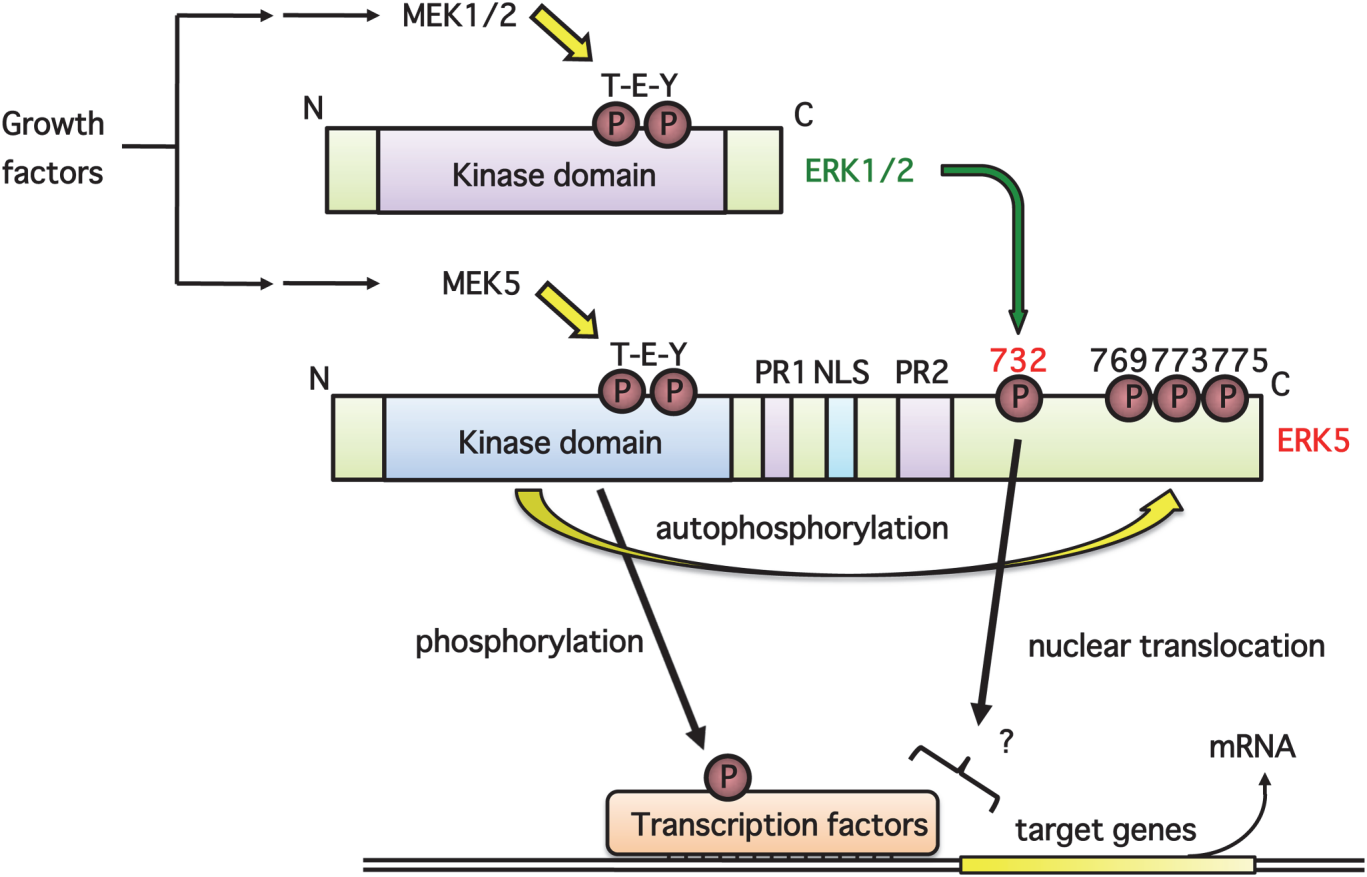
Putative cross-talk mechanism between ERK5 and ERK1/2. **(1)** Growth factors such as NGF and EGF promote phosphorylation of ERK1/2 and ERK5 at their TEY motifs through MEK1/2 and MEK5, respectively, then their kinase activity increases rapidly upon phosphorylation. (**2)** Activated ERK5 autophosphorylates its C-terminal residues including Ser769/773/775. **(3)** Activated ERK1/2 phosphorylates the C-terminal of ERK5 at Thr732; this phosphorylation event is essential for ERK5 nuclear localization and ERK5-dependent transcription via an unknown mechanism.

In this study, we demonstrate that ERK1/2 phosphorylates ERK5 at Thr732 upon growth factor stimulation, and that this phosphorylation is necessary for ERK5 nuclear localization and ERK5-dependent transcription. A putative signaling pathway is shown in Fig. 7. We showed ERK5 autophosphorylation at Thr732 and Ser769/773/775 using phospho-specific antibodies (Fig. 1). Interestingly, regardless of the phosphorylation status of the ERK5 TEY motif or ERK5 kinase activity, ERK5 phosphorylation at Thr732, but not at Ser769/773/775, was induced by ERK1/2 activated by oncogenic RAS signaling (Fig. 3). ERK5 phosphorylation at Thr732 induced by growth factors that activate both ERK5 and ERK1/2 signaling was also ERK1/2-dependent (Fig. 4). This phosphorylation event was involved in the nuclear retention of ERK5 and MEF2 activation (Figs. 5 and 6), indicating that ERK1/2 activity is required for maximum ERK5 activity. However, the role of phosphorylation at Thr732 by ERK1/2 remains unclear. This site may promote the nuclear translocation of ERK5 (i.e. nuclear localization or nuclear export of ERK5 may be promoted or inhibited by Thr732 phosphorylation, respectively) or its binding to transcription factors, resulting in their efficient phosphorylation. Indeed, it has been shown that multiple serine and threonine residues in the ERK5 C-terminal, including Ser706, Thr732, Ser753 and Ser773, are phosphorylated in a cyclin-dependent kinase 1-dependent manner that does not involve the canonical MEK5-dependent route. These phosphorylation events are necessary for the retention of ERK5 in the nucleus and activation of the Nur77 promoter [33]. In addition, mimetics of the ERK5 C-terminal phosphorylated at Thr732 and Ser769/773/775 can promote activity of the AP-1 and MEF2C transcription factors independently of ERK5 kinase activity [4].

Endogenous ERK5 co-immunoprecipitated with ERK2, although, surprisingly, the interaction between ERK5 and ERK2 (possibly ERK1 as well) did not change in response to NGF under our experimental conditions (Fig. 2). It has been shown that ERK5 and ERK2 form homodimers/oligomers. However, it is unclear whether ERK5 can also form heterodimers/oligomers with ERK2 while other ERK5 and ERK2 molecules form homodimers/oligomers, respectively, or whether hetero-oligomers composed of ERK5 and ERK2 homodimers are present as well. Furthermore, it is currently unclear if the ability of ERK5 and ERK1/2 to form complexes is dependent on their phosphorylation status. In addition, the interaction between ERK5 and ERK1/2 was indirect under our experimental conditions (data not shown), suggesting that an unidentified protein mediates the interaction between these ERKs.

It is thought that the interaction between ERK5 and ERK1/2 is essential for ERK1/2-mediated phosphorylation of ERK5 at Thr732, nuclear retention and the enhancement of MEF2 transcriptional activity. Drastic ERK5 conformational changes elicited by phosphorylation by MEK5 allow exposure of its constitutively active nuclear localization signal (509–539) and inhibition of its nuclear export signal by dissociation of intramolecular binding between the ERK5 N and C termini. This results in a predominantly nuclear localization [2,35,36,37]. In contrast, ERK1/2 contains no obvious nuclear localization signal or nuclear export signal. In resting cells, MEK1/2, which has a nuclear export signal, anchors ERK1/2 in the cytoplasm. Upon stimulation, ERK1/2 dissociates from MEK1/2 and translocates to the nucleus. Although it has been suggested that ERK1/2 nuclear translocation is mediated by passive diffusion of monomers, active transport of dimers and an interaction with nuclear pore complexes, the underlying mechanisms remain unclear.

Because MEF2 activation by EGF was significantly inhibited by U0126 and overexpression of ER–32A (Fig. 6), we inferred that ERK1/2-dependent phosphorylation of ERK5 at Thr732 is important for MEF2 activation by ERK5. Although it is well known that ERK5, but not ERK1/2, directly phosphorylates MEF2C at Ser387, which is critical for transcriptional activation, it has been demonstrated that phosphorylation of Ser192 by p90 ribosomal S6 kinase 2, a downstream effector of ERK1/2, is also essential for the transcriptional activity of MEF2C [38]. Hence, Fig. 6A suggests either of two possibilities: (1) ERK1/2 phosphorylation of ERK5 at Thr732 or (2) ERK1/2-dependent phosphorylation of MEF2. However, the result presented in Fig. 6B supports the former. ERK5 phosphorylated at Thr732 can retain nuclear localization to a greater extent than the unphosphorylated form (Fig. 5), which may, in turn, contribute to MEF2C activation.

We found that the binding between ERK5 and ERK1/2 was indirect, and we did not determine whether ERK5 Thr732 phosphorylation by ERK1/2 was direct or indirect using *in vitro* kinase assay. However, because the phosphorylation site has the minimal consensus sequence for phosphorylation by ERK1/2 (i.e. Ser/Thr-Pro) [39], we surmise that ERK1/2 interacts with ERK5 and phosphorylates ERK5 at Thr732 directly. The threonine residue equivalent of human ERK5 Thr732 is conserved among numerous species, including mouse, rat and Xenopus, suggesting that phosphorylation at this amino acid plays a critical functional role in metazoa.

Reduced ERK5 electrophoretic mobility (phosphorylation shift) is often regarded as an index of ERK5 activation. However, the phosphorylation status of the ERK5 C-terminal, including Thr732, influences ERK5 electrophoretic mobility regardless of ERK5 phosphorylation at its TEY motif, as shown in Fig. 3. Thus, it is inappropriate to regard the ERK5 band shift as an index of ERK5 kinase activity. In addition, ERK5 immunocomplex kinase assays have to be determined more carefully because ERK1/2 co-immunoprecipitates with ERK5 (Fig. 2). Consequently, an ERK5-specific substrate must be used for kinase assays *in vitro*, and it is necessary to carefully adjust immunoprecipitation conditions, including salt and detergent concentrations, to allow the immunoprecipitation of ERK5 but disturbing ERK2 binding.

In conclusion, we demonstrate a new cross-talk mechanism between ERK5 and ERK1/2 in which phosphorylation of ERK5 at Thr732 is induced by ERK1/2, and showed that this phosphorylation event is required for ERK5 nuclear localization and MEF2C activation. Further studies are necessary to clarify how phosphorylation status influences ERK5 structure and to elucidate the mechanisms underlying MEF2C activation.
